# Sex-stratified patterns of emergency cardiovascular admissions prior and during the COVID-19 pandemic

**DOI:** 10.1038/s41598-023-44400-3

**Published:** 2023-10-20

**Authors:** Piotr Gajewski, Mikołaj Błaziak, Szymon Urban, Mateusz Garus, Frieder Braunschweig, Daniel Caldeira, Antoni Gawor, John P. Greenwood, Mateusz Guzik, Frank R. Halfwerk, Gracjan Iwanek, Michał Jarocki, Maksym Jura, Małgorzata Krzystek-Korpacka, Łukasz Lewandowski, Lars H. Lund, Michał Matysiak, Fausto Pinto, Jakub Sleziak, Weronika Wietrzyk, Mateusz Sokolski, Jan Biegus, Piotr Ponikowski, Robert Zymliński

**Affiliations:** 1 Institute of Heart Diseases, Wroclaw Medical University, Wrocław, Poland; 2 Department of Medicine, Department of Cardiology, Karolinska Institutet, Karolinska University Hospital, Stockholm, Sweden; 3 Cardiovascular da Universidade de Lisboa – CCUL (CCUL@RISE), CEMBE, Faculdade de Medicina, Universidade de Lisboa, Lisbon, Portugal; 4 Cardiology Department, Hospital Universitário de Santa Maria (CHLN), Centro Académico de Medicina de Lisboa (CAML), Lisbon, Portugal; 5 Student Scientific Organization, Institute of Heart Diseases, Wroclaw Medical University, Wrocław, Poland; 6 Leeds University and Leeds Teaching Hospitals NHS Trust, Leeds,, UK; 7 Thorax Center Twente, Medisch Spectrum Twente, Enschede, The Netherlands; 8 Department of Medical Biochemistry, Wroclaw Medical University, Wrocław, Poland

**Keywords:** Cardiology, Medical research, Acute coronary syndromes, Heart failure

## Abstract

The COVID-19 pandemic has had a significant impact on global public health, with long-term consequences that are still largely unknown. This study aimed to assess the data regarding acute cardiovascular hospital admissions in five European centers before and during the pandemic. A multicenter, multinational observational registry was created, comparing admissions to the emergency departments during a 3-months period in 2020 (during the pandemic) with the corresponding period in 2019 (pre-pandemic). Data on patient demographics, COVID-19 test results, primary diagnosis, comorbidities, heart failure profile, medication use, and laboratory results were collected. A total of 8778 patients were included in the analysis, with 4447 patients in 2019 and 4331 patients in 2020. The results showed significant differences in the distribution of cardiovascular diseases between the two years. The frequency of pulmonary embolism (PE) increased in 2020 compared to 2019, while acute heart failure (AHF) and other cardiovascular diseases decreased. The odds of PE incidence among hospitalized patients in 2020 were 1.316-fold greater than in 2019. The incidence of AHF was 50.83% less likely to be observed in 2020, and the odds for other cardiovascular diseases increased by 17.42% between the 2 years. Regarding acute coronary syndrome (ACS), the distribution of its types differed between 2019 and 2020, with an increase in the odds of ST-segment elevation myocardial infarction (STEMI) in 2020. Stratification based on sex revealed further insights. Among men, the incidence of AHF decreased in 2020, while other cardiovascular diseases increased. In women, only the incidence of STEMI showed a significant increase. When analyzing the influence of SARS-CoV-2 infection, COVID-positive patients had a higher incidence of PE compared to COVID-negative patients. COVID-positive patients with ACS also exhibited symptoms of heart failure more frequently than COVID-negative patients. These findings provide valuable information on the impact of the COVID-19 pandemic on acute cardiovascular hospital admissions. The increased incidence of PE and changes in the distribution of other cardiovascular diseases highlight the importance of monitoring and managing cardiovascular health during and post pandemic period. The differences observed between sexes emphasize the need for further research to understand potential sex-specific effects of COVID-19 on cardiovascular outcomes.

## Introduction

The coronavirus disease 2019 (COVID-19) pandemic has had a profound impact on global public health. The long-term consequences of COVID-19 remain unknown in terms of future morbidity and mortality, impact on health service provision and in particular, patient physical and emotional health in relation to long COVID.

The analysis of changes in the distribution of certain medical events during the pandemic may provide insight into the implications of COVID-19 and, likewise, public health restrictions. Unfortunately, this task is hindered due to the limitation of access to national public medical care records in many healthcare systems, and the urgent reorganization of medical services at the start of the pandemic^1^. The aforementioned factors could have potentially influenced both the numbers and disease types/severity of patients admitted to the hospital^2^, and also by patient choices made due to anxiety about leaving isolation at home and seeking acute care. The aim of this study was to assess data regarding acute cardiovascular hospital admissions in five European centers before and during the pandemic.

## Materials and methods

### Study design

A multicenter, multinational observational registry was created based on admissions to the emergency departments (ED) throughout 3 months (October–December) in 2020, during the European stages of the COVID-19 pandemic. The data was compared to the corresponding 3-month period in the year 2019 (pre-pandemic).

No exclusion criteria were applied; all patients admitted to the emergency department during the aforementioned period were comprehensively incorporated into the registry. Five European centers: including the Institute of Heart Diseases of Wroclaw Medical University from Poland, Karolinska Institutet, Department of Cardiology from Sweden, Cardiovascular da Universidade de Lisbona from Portugal, Leeds University and Leeds Teaching Hospitals from UK and Thorax Center Twente from the Netherlands participated in the registry. All centers contributed data on acute admissions to the ED in 2019 and 2020. The following data were collected: sex, age, BMI, and the result of reverse transcription-polymerase chain reaction tests from upper airway swabs for SARS-CoV-2 (if available).

In patients admitted to the hospital cardiology departments, the following data were collected: primary diagnosis, comorbidity, the profile of heart failure, used drugs and laboratory results. The primary diagnosis was classified into five major categories: ‘Acute coronary syndrome (ACS)’, ‘Acute heart failure (AHF)’, ‘Pulmonary embolism (PE)’, ‘Arrhythmia’ and ‘Others’. The ‘Others’ label was applied when none of the predetermined diagnoses was observed.

Patients admitted with ACS were categorized as: ‘Unstable angina (UA)’, ‘ST-segment elevation myocardial infarction (STEMI)’ or ‘non-ST-segment elevation myocardial infarction (NSTEMI)’. ACS was defined according to the Fourth Universal Definition of Myocardial Infarction^3^. AHF was diagnosed according to the guidelines for the diagnosis and treatment of acute and chronic heart failure^4^. PE was diagnosed according to the guidelines of acute pulmonary embolism^5^. Arrhythmia on admission was classified as: ‘Atrial’, ‘Ventricular’, ‘Heart block or bradycardia’. Ventricular arrythmias incorporated ventricular fibrillation and ventricular tachycardia^6^. Atrial arrhythmias included atrial fibrillation, atrial flutter, tachycardia with narrow QRS and supraventricular tachycardia with wide QRS^7^. Bradycardia was diagnosed when symptomatic and included heart block (second- and third-degree).

*Comorbidities* were classified into five categories: ‘Atrial fibrillation/flutter’, ‘chronic kidney disease’, ‘Diabetes’, 'Arterial hypertension’ and ‘Cardiac resynchronization therapy (CRT)/implantable cardioverter-defibrillator (ICD) therapy’.

*Profile of heart failure* was assessed by: etiology, ejection fraction, cause of deterioration, time of presentation of symptoms, clinical profile and used drugs.

*Laboratory* results included: N-terminal prohormone of brain natriuretic peptide (NT-proBNP), brain natriuretic peptide (BNP), troponin, creatinine and haemoglobin levels on admission.

Records were obtained from the individual centres' databases. Preprocessing and management of the final registry were provided by Wroclaw Medical University, Wrocław, Poland. The complete registry was checked for missing data or any input errors. The principal investigator from each center was responsible for the accuracy and quality of anonymised shared data. The study protocol was approved by the local ethics committees (Bioethics Committee of Wroclaw Medical University, Poland and Medical Ethical Research Committees United, The Netherlands) and was conducted in accordance with the Declaration of Helsinki. Since the study is retrospective, the need for informed consent was waived by ethics committees: Bioethics Committee of Wroclaw Medical University, Poland and Medical Ethical Research Committees United, The Netherlands.

### Data analysis

Data preprocessing was performed using Python 3.8.10 (used packages: numpy 1.21.6, pandas 1.4.2) in Visual Studio Code 1.68.0. Statistical analyses were performed in R 3.6.1 (used packages: tidyverse 1.3.1, samplesizeCMH 0.0.0, vcdExtra 0.8–0) or with STATISTICA 13.3 on the license of Wroclaw Medical University. The significance of the difference in counts was assessed with χ^2^ test, based on Pearson's χ^2^ or Yates' χ^2^ statistic (if any of the expected counts was < 5). Additionally, to provide more accurate information on the odds ratios (ORs) computed in the whole population sample, a generalized Cochran-Mantel–Haenszel test was performed in the context of data stratification according to sex. In other cases, the U Mann–Whitney test was performed. A *p*-value < 0.05 was deemed significant.

## Results

Five European cardiac centers participated in the registry, and data from 8778 patients (mean age 67 ± 16 years, 62% male) were acquired and analyzed. The registry comprised predominantly Caucasian patients from the European population. Data included 4447 patients admitted to emergency departments in 2019 and 4331 patients in 2020. During the study periods, 2022 patients (23%) were tested for the SARS-CoV-2 infection and in 180 patients (2% of the total population sample) the tests results were positive. The mean age of COVID-19 infected patients did not differ from the general study population. The SARS-CoV-2 positive patients were predominantly male (67%).

### The difference in prevalence of selected cardiovascular diseases in years 2019–2020

**Figure 1:**
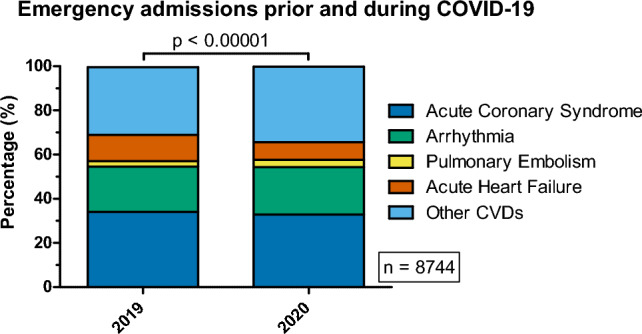
Distribution of selected cardiovascular diseases associated with emergency admissions prior (2019) and during the COVID-19 pandemic (2020).

**Figure 2:**
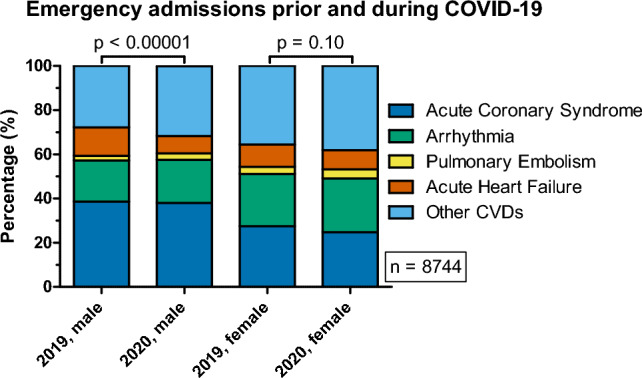
Distribution of selected cardiovascular diseases associated with emergency admissions prior (2019) and during the COVID-19 pandemic (2020) after stratification by sex.

Three types of analyses were performed to assess the difference in the prevalence of ACS, AHF, arrhythmia, PE and 'Other CVDs' between years 2019 and 2020. All analyses were performed on the whole population and, separately, in context of different sex. The first analysis was performed to assess differences in disease distribution (Table 1, Fig. 1). The second, to check whether the odds ratios for incidence of these diseases differed between the years 2019 and 2020, after stratification based on sex (Table 2, Fig. 2). The last analysis was performed to assess whether the distribution of the subtypes of selected diseases and/or their coincidence with symptoms of heart failure varied in time (Table 3).

**Table 1:** Time-related frequencies of the selected CVD categories.

Strata	Year: 2019	Year: 2020	Year: 2019	Year: 2020	χ^2^	*p*
	Count (n)	Count (n)	Frequency (%)	Frequency (%)		
Overall distribution (regardless of sex, N = 8744)						
ACS	1530 (1496.24)	1414 (1447.76)	34.43	32.88	44.64	< 0.00001
Arrhythmia	911 (931.09)	921 (900.91)	20.50	21.42		
PE	114 (131.12)	144 (126.88)	2.57	3.35		
AHF	527 (446.74)	352 (432.26)	11.86	8.19		
Other CVDs	1362 (1438.81)	1469 (1392.19)	30.65	34.16		
Subset distribution (sex: female, N = 3333)						
ACS	454 (431.76)	415 (437.24)	27.42	24.75	7.69	0.1037
Arrhythmia	393 (398.47)	409 (403.53)	23.73	24.39		
PE	53 (60.12)	68 (60.88)	3.20	4.05		
AHF	168 (155.51)	145 (157.49)	10.14	8.65		
Other CVDs	588 (610.13)	640 (617.87)	35.51	38.16		
Subset distribution (sex: male, N = 5411)						
ACS	1076 (1069.14)	999 (1005.86)	38.59	38.09	42.25	< 0.00001
Arrhythmia	518 (530.7)	512 (499.3)	18.58	19.52		
PE	61 (70.59)	76 (66.41)	2.19	2.90		
AHF	359 (291.63)	207 (274.37)	12.88	7.89		
Other CVDs	774 (825.94)	829 (777.06)	27.76	31.61		

Numbers in ‘()’ brackets indicate expected (estimated) counts.

**Table 2:** Time-related differences in incidence of selected CVDs.

Overall distribution (regardless of sex, N = 8744)															
Variable	Class	Year: 2019	Year: 2020	Year: 2019	Year: 2020	No stratification corrections					Generalized CMH (adjusted for stratification by sex)				
		count (n)	count (n)	frequency (%)	frequency (%)	χ^2^	p	OR	OR {− 95% CI}	OR {95% CI}	χ^2^	p	OR	OR {− 95% CI}	OR {95% CI}
ACS	ACS	1530 (1496.24)	1414 (1447.76)	34.43	32.88	2.34	0.1265	–	–	–	1.76	0.1842	–	–	–
	No ACS	2914 (2947.76)	2886 (2852.24)	65.57	67.12										
Arrhythmia	Arrhythmia	911 (9319)	921 (900.91)	20.50	21.42	1.11	0.2910	–	–	–	0.92	0.3385	–	–	–
	No arrhythmia	3533 (3512.91)	3379 (3399.09)	79.50	78.58										
PE	PE	114 (131.12)	144 (126.88)	2.57	3.35	4.69	**0.0304**	**1.316**	1.026	1.689	4.47	**0.0346**	**1.308**	1.019	1.678
	No PE	4330 (4312.88)	4156 (4173.12)	97.43	96.65										
AHF	AHF	527 (446.74)	352 (432.26)	11.86	8.19	32.60	** < 0.00001**	**0.663**	0.575	0.764	32.29	** < 0.00001**	**0.664**	0.576	0.765
	No AHF	3917 (3997.26)	3948 (3867.74)	88.14	91.81										
Other CVDs	Other CVDs	1362 (1438.81)	1469 (1392.19)	30.65	34.16	12.33	**0.0005**	**1.174**	1.073	1.284	11.53	**0.0007**	**1.169**	1.068	1.278
	ACS/arrhythmia/PE/AHF	3082 (3005.19)	2831 (2907.81)	69.35	65.84										

Subset distribution (sex: female, N = 3333)															
Variable	Class	Year: 2019	Year: 2020	Year: 2019	Year: 2020	χ^2^	*p*	OR	OR {− 95% CI}	OR {95% CI}					
		Count (n)	Count (n)	Frequency (%)	Frequency (%)										
ACS	ACS	454 (431.76)	415 (437.24)	27.42	24.75	3.08	0.0793	–	–	–					
	No ACS	1202 (1224.24)	1262 (1239.76)	72.58	75.25										
Arrhythmia	Arrhythmia	393 (398.47)	409 (403.53)	23.73	24.39	0.20	0.6573	–	–	–					
	No arrhythmia	1263 (1257.53)	1268 (1273.47)	76.27	75.61										
PE	PE	53 (60.12)	68 (60.88)	3.20	4.05	1.74	0.1873	1.278	0.887	1.843					
	No PE	1603 (1595.88)	1609 (1616.12)	96.80	95.95										
AHF	AHF	168 (155.51)	145 (157.49)	10.14	8.65	2.20	0.1381	0.838	0.664	1.059					
	No AHF	1488 (1500.49)	1532 (1519.51)	89.86	91.35										
Other CVDs	Other CVDs	588 (610.13)	640 (617.87)	35.51	38.16	2.53	0.1120	1.121	0.974	1.291					
	ACS/arrhythmia/PE/AHF	1068 (1045.87)	1037 (1059.13)	64.49	61.84										

Subset distribution (sex: male, N = 5411)															
Variable	Class	Year: 2019	Year: 2020	Year: 2019	Year: 2020	χ^2^	p	OR	OR {− 95% CI}	OR {95% CI}					
		Count (n)	Count (n)	Frequency (%)	Frequency (%)										
ACS	ACS	1076 (1069.14)	999 (1005.86)	38.59	38.09	0.15	0.7010	–	–	–					
	No ACS	1712 (1718.86)	1624 (1617.14)	61.41	61.91										
Arrhythmia	Arrhythmia	518 (530.7)	512 (499.3)	18.58	19.52	0.77	0.3787	–	–	–					
	No arrhythmia	2270 (2257.3)	2111 (2123.7)	81.42	80.48										
PE	PE	61 (70.59)	76 (66.41)	2.19	2.90	2.76	0.9684	1.334	0.948	1.876					
	No PE	2727 (2717.41)	2547 (2556.59)	97.81	97.10										
AHF	AHF	359 (291.63)	207 (274.37)	12.88	7.89	35.86	** < 0.00001**	**0.580**	0.484	0.694					
	No AHF	2429 (2496.37)	2416 (2348.63)	87.12	92.11										
Other CVDs	Other CVDs	774 (825.94)	829 (777.06)	27.76	31.61	9.57	**0.0020**	**1.202**	1.070	1.351					
	ACS/arrhythmia/PE/AHF	2014 (1962.06)	1794 (1845.94)	72.24	68.39										

Numbers in ‘()’ brackets indicate expected (estimated) counts. Significant odds ratios (ORs) are marked in bold.

**Table 3:** Time-related differences in CVD subtypes and CVD-related incidence of heart failure.

Overall distribution (regardless of sex)																	
Variable	Class	Year: 2019	Year: 2020	Year: 2019	Year: 2020	No stratification corrections							Generalized CMH (adjusted for stratification by sex)				
		Count (n)	Count (n)	Frequency (%)	Frequency (%)	χ^2^	*p*	OR	OR {− 95% CI}	OR {95% CI}	*p* (each class)	χ^2^	*p*	OR	OR {− 95% CI}	OR {95% CI}	*p* (each class)
ACS: type	NSTEMI	607 (574.2)	498 (530.8)	42.09	37.36	18.73	**0.0001**	**0.820**	0.704	0.956	**0.0109**	18.63	**0.0001**	**0.821**	0.705	0.956	**0.0112**
	STEMI	583 (638.64)	646 (590.36)	40.43	48.46			**1.385**	1.192	1.610	**0.00002**			**1.385**	1.191	1.610	** < 0.00002**
	Unstable angina	252 (229.16)	189 (211.84)	17.48	14.18			**0.780**	0.635	0.958	**0.0176**			**0.780**	0.636	0.958	**0.0178**
ACS: heart failure	Symptoms present	216 (214.69)	196 (197.31)	14.95	14.76	0.020	0.8888	–	–	–	–	0.016	0.8981	–	–	–	–
	No symptoms	1229 (1230.31)	1132 (1130.69)	85.05	85.24												
Arrhythmia: type	Atrial	581 (577.41)	566 (569.59)	65.50	64.69	1.77	0.4122	–	–	–	–	1.81	0.4054	–	–	–	–
	Ventricular	110 (103.7)	96 (102.3)	12.40	10.97												
	Heart block, bradycardia	196 (205.89)	213 (203.11)	22.10	24.34												
Arrhythmia: heart failure	Symptoms present	131 (142.44)	159 (147.56)	26.15	30.64	2.52	0.1122	–	–	–	–	2.62	0.1058	–	–	–	–
	No symptoms	370 (358.56)	360 (371.44)	73.85	69.36												
PE: heart failure	Symptoms present	15 (13.98)	17 (18.02)	14.02	12.32	0.15	0.6953	–	–	–	–	0.13	0.7182	–	–	–	–
	No symptoms	92 (93.02)	121 (119.98)	85.98	87.68												
AHF: type	De novo	55 (63.5)	47 (38.5)	21.65	30.52	4.02	0.0450	1.589	1.009	2.504	–	3.705	0.0542	–	–	–	–
	CHF decompensation	199 (190.5)	107 (115.5)	78.35	69.48												

Subset distribution (sex: female)																	
Variable	Class	Year: 2019	Year: 2020	Year: 2019	Year: 2020	χ^2^	*p*	OR	OR {− 95% CI}	OR {95% CI}	*p* (each class)						
		count (n)	count (n)	frequency (%)	frequency (%)												
ACS: type	NSTEMI	189 (180.96)	156 (164.04)	44.16	40.21	4.31	0.1160	0.850	0.644	1.123	0.2539						
	STEMI	162 (176.24)	174 (159.76)	37.85	44.85			1.335	1.009	1.766	0.0427						
	Unstable angina	77 (70.81)	58 (64.19)	17.99	14.95			0.801	0.552	1.163	0.2431						
ACS: heart failure	Symptoms present	71 (71.03)	64 (63.97)	16.78	16.80	< 0.0001	0.9961	–	–	–	–						
	No symptoms	352 (351.97)	317 (317.03)	83.22	83.20												
Arrhythmia: type	Atrial	282 (276.88)	279 (284.12)	73.82	71.17	2.47	0.2914	–	–	–	–						
	Ventricular	35 (32.08)	30 (32.92)	9.16	7.65												
	Heart block, bradycardia	65 (73.04)	83 (74.96)	17.02	21.17												
Arrhythmia: heart failure	Symptoms present	46 (50.74)	58 (53.26)	20.81	25.00	1.12	0.2897	–	–	–	–						
	No symptoms	175 (170.26)	174 (178.74)	79.19	75.00												
PE: heart failure	Symptoms present	4 (4.69)	7 (6.31)	8.16	10.61	0.14	0.9046	–	–	–	–						
	No symptoms	45 (44.31)	59 (59.69)	91.84	89.39												
AHF: type	CHF decompensation	65 (60.96)	38 (42.04)	74.71	63.33	2.19	0.1387	–	–	–	–						
	De novo	22 (26.04)	22 (17.96)	25.29	36.67												

Subset distribution (sex: male)																	
Variable	Class	Year: 2019	Year: 2020	Year: 2019	Year: 2020	χ^2^	*p*	OR	OR {− 95% CI}	OR {95% CI}	*p* (each class)						
		Count (n)	Count (n)	Frequency (%)	freqUency (%)												
ACS: type	NSTEMI	418 (393.38)	342 (366.62)	41.22	36.19	14.43	**0.0007**	**0.809**	0.674	0.970	**0.0222**						
	STEMI	421 (462.23)	472 (430.77)	41.52	49.95			**1.406**	1.176	1.680	**0.0002**						
	Unstable angina	175 (158.39)	131 (147.61)	17.26	13.86			**0.772**	0.603	0.987	**0.0386**						
ACS: heart failure	Symptoms present	145 (143.78)	132 (133.22)	14.19	13.94	0.03	0.8737	–	–	–							
	No symptoms	877 (878.22)	815 (813.78)	85.81	86.06												
Arrhythmia: type	Atrial	299 (299.52)	287 (286.48)	59.21	59.42	0.33	0.8461	–	–	–							
	Ventricular	75 (72.07)	66 (68.93)	14.85	13.66												
	Heart block, bradycardia	131 (133.41)	130 (127.59)	25.94	26.92												
Arrhythmia: heart failure	Symptoms present	85 (91.85)	101 (94.15)	30.36	35.19	1.50	0.2203	–	–	–							
	No symptoms	195 (188.15)	186 (192.85)	69.64	64.81												
PE: heart failure	Symptoms present	11 (9.37)	10 (11.63)	18.97	13.89	0.61	0.4343	–	–	–							
	No symptoms	47 (48.63)	62 (60.37)	81.03	86.11												
AHF: type	CHF decompensation	134 (129.89)	69 (73.11)	80.24	73.40	1.63	0.2023	–	–	–							
	De novo	33 (37.11)	25 (20.89)	19.76	26.60												

Numbers in ‘()’ brackets indicate expected (estimated) counts. Significant odds ratios (ORs) are marked in bold.

#### Observations on the total, unstratified population

In the total population studied, there were significant differences (Table 1, Fig. 1) in the distributions of the analyzed CVDs (*p* < 0.00001), as a result of significant increases in the frequency of PE (*p* ≈ 0.0304), and decreases in AHF (*p* < 0.00001) and ‘Other CVDs’ (*p* ≈ 0.0005) from 2019 to 2020. The frequencies of PE in 2020 and 2019 were: 3.35% and 2.57%, respectively. The odds ratio of the incidence of PE among hospitalized patients in 2020 were 1.316-fold greater compared to the odds in 2019 (OR 1.315, 95% CI 1.026–1.689). Interestingly, the frequency of AHF dropped from 2019 to 2020 (11.86% vs. 8.19%). The incidence of AHF among hospitalized patients was 50.83% less likely (OR = 0.663) to be observed in 2020, compared to 2019. The odds for the incidence of Other CVDs increased by 17.42% (OR = 1.1742) between the years 2019 and 2020 (frequencies: 30.65% and 34.16%, respectively).

Although the incidence of ACS did not differ between 2019 and 2020 (*p* ≈ 0.1265), an interesting observation emerged upon analysis of the distribution of its types: NSTEMI, STEMI and unstable angina, between 2019 and 2020 (*p* ≈ 0.0001). The odds of incidence of NSTEMI and unstable angina were lower in 2020, compared to 2019 (*p*-values: 0.0109, 0.0176; ORs: 0.8204, 0.7802 for the incidence of NSTEMI, unstable angina, respectively). Conversely, the odds for the incidence of STEMI were approximately 38.55% higher in 2020 (OR = 1.3855), compared to 2019 (*p* ≈ 0.00002). De novo AHF was more frequent in 2020, compared to 2019 (OR = 1.589, *p* ≈ 0.0450), although the significance of this observation was ambiguous (as described in subsection "Data analysis".). The frequencies are shown in Table 3.

#### The influence of stratification based on sex on the observations in the total population of hospitalized patients

When the whole population sample was stratified according to sex (Table 2, Fig. 2), a significant difference in the distribution of the aforementioned diseases was observed in men (*p* < 0.00001). In women, this difference was not significant (*p* ≈ 0.1037). Interestingly neither men nor women subpopulations showed a significant increase in PE incidence, as was observed in the whole population. Among men, the incidence of AHF was 72.41% less likely (OR = 0.580, *p* < 0.00001) in 2020 (frequency: 7.89%), compared to 2019 (12.88%). A higher incidence of Other CVDs was observed among men in 2020 (frequency: 31.61%), compared to 2019 (frequency: 27.76%). The odds for the incidence of these diseases in men were 20.24% greater (OR = 1.2024, *p* ≈ 0.0020) in 2020, compared to 2019. All the previously described observations regarding the incidence of the analyzed CVDs remained statistically significant after accounting for the stratification. The adjusted odds ratios describing the incidence of PE, AHF and Other CVDs are similar to those computed without accounting for stratification (Table 2).

Stratification revealed that STEMI, NSTEMI and unstable angina were differently distributed among men (*p* ≈ 0.0007), but not in women (*p* ≈ 0.1160). Nevertheless, analysis of the incidence of each ACS type separately revealed significant differences in both sexes. In men, the incidence of NSTEMI and unstable angina decreased in 2020, compared to 2019 (*p*-values: 0.0222, 0.0386; ORs: 0.8087, 0.7716 for NSTEMI, unstable angina, respectively). The frequency of STEMI increased by 8.43 percentage points (40.56% increase in odds, OR = 1.4056) in men between the years 2019 and 2020. In women, only the differences in the incidence of STEMI were significant (*p* ≈ 0.0427), accounting for an increase in the odds for STEMI incidence by 33.51% (OR = 1.3351) between years 2019 and 2020 (frequencies: 37.85% and 44.85% in years: 2019 and 2020, respectively). This finding in women should be interpreted with caution, since the global test for differences in distribution of the types of ACS showed lack of significance (*p* ≈ 0.1160). The overall odds ratio (describing the entire population sample after accounting for stratification) for the incidence of ACS types between the years 2019 and 2020 is shown in Table 3.

It should be emphasized, that the OR describing the incidence of de novo AHF compared to the incidence of CHF decompensation AHF between the years 2019 and 2020, was insignificant after adjusting for stratification based on sex (*p* ≈ 0.0542). Therefore, the underlying hypothesis is not likely to be true in the general population (and does not hold in male and female subpopulations either).

### The influence of SARS-CoV2 on the prevalence of selected cardiovascular diseases in 2020

Data analysis was performed in a similar manner as was explained in “The difference in prevalence of selected cardiovascular diseases in years 2019–2020” Section, although COVID-19 incidence was analyzed as a grouping factor. All the data described in this section was gathered in 2020 during the outbreak of SARS-CoV2. Only positive and negative test results for SARS-CoV2 were considered in this analysis (COVID-positive and COVID-negative).

#### Observations on the total, unstratified population

The distribution of all the analyzed CVD categories differed between COVID-positive and COVID-negative patients (*p *≈ 0.0001). An approximately 4.61-fold increase (OR = 4.6091, *p* < 0.00001) of the odds for the incidence of PE was observed in COVID-positive patients (frequency: 6.70%), compared to COVID-negative (frequency: 1.54%). More information is shown in Table 4.

**Table 4:** The distribution of all the analyzed CVD in the context of the SARS-Cov-2 test.

Strata	SARS-Cov-2: negative	SARS-Cov-2: positive	SARS-Cov-2: negative	SARS-Cov-2: positive	χ^2^	*p*
	Count (n)	Count (n)	Frequency (%)	Frequency (%)		
Overall distribution (regardless of sex, N = 2003)						
ACS	815 (805.0)	69 (79.0)	44.68	38.55	24.95	0.0001
Arrhythmia	327 (322.36)	27 (31.64)	17.93	15.08		
PE	28 (36.43)	12 (3.57)	1.54	6.70		
AHF	155 (158.45)	19 (15.55)	8.50	10.61		
Other CVDs	499 (501.76)	52 (49.24)	27.36	29.05		
Subset distribution (sex: female, N = 767)						
ACS	263 (256.62)	15 (21.38)	37.15	25.42	21.34	0.0003
Arrhythmia	154 (154.15)	13 (12.85)	21.75	22.03		
PE	14 (18.46)	6 (1.54)	1.98	10.17		
AHF	58 (62.77)	10 (5.23)	8.19	16.95		
Other CVDs	219 (216.0)	15 (18.0)	30.93	25.42		
Subset distribution (sex: male, N = 1236)						
ACS	552 (547.17)	54 (58.83)	49.46	45.00	12.46	0.0142
Arrhythmia	173 (168.84)	14 (18.16)	15.50	11.67		
PE	14 (18.06)	6 (1.94)	1.25	5.00		
AHF	97 (95.71)	9 (10.29)	8.69	7.50		
Other CVDs	280 (286.22)	37 (30.78)	25.09	30.83		

Numbers in ‘()’ brackets indicate expected (estimated) counts.

Interestingly, COVID-positive patients with ACS were reported to show symptoms of heart failure more frequently, compared to COVID-negative patients (*p* ≈ 0.0004, frequencies: 33.33% vs. 15.76%). The computed odds for the incidence of symptoms of heart failure among COVID-positive patients with ACS was approximately 2.67fold greater (OR = 2.6724) than the odds observed in COVID-negative patients.

#### Sex based analysis of the whole study population

Differences in the distribution of analyzed CVDs were significant in both female (*p* ≈ 0.0003) and male (*p* ≈ 0.0142) subpopulations. Both men and women showed a higher incidence of PE in the context of positive SARS-CoV2 test results. The increase in the odds for PE incidence was more pronounced in women, compared to men (*p*-values: 0.0002, 0.0020; ORs: 5.6119, 4.1429 for women and men, respectively), as the frequency of PE in COVID-positive women was higher by 8.19 percentage points (frequencies: 10.19%, 1.98% in COVID-positive and COVID-negative females, respectively). The ORs (adjusted for stratification) describing the whole population sample are shown in Table 5).

**Table 5:** Distribution of CVD types in the context of SARS-Cov-2 test and sex.

Overall distribution (regardless of sex, N = 2003)															
Variable	Class	SARS-Cov-2: negative	SARS-Cov-2: positive	SARS-Cov-2: negative	SARS-Cov-2: positive	No stratification corrections					Generalized CMH (adjusted for stratification by sex)				
		Count (n)	Count (n)	Frequency (%)	Frequency (%)	χ^2^	p	OR	OR {− 95% CI}	OR {95% CI}	χ^2^	p	OR	OR {− 95% CI}	OR {95% CI}
ACS	ACS	815 (805.0)	69 (79.0)	44.68	38.55	2.49	0.1147	–	–	–	3.17	0.0750	–	–	–
	No ACS	1009 (1019.0)	110 (100.0)	55.32	61.45										
Arrhythmia	Arrhythmia	327 (322.36)	27 (31.64)	17.93	15.08	0.91	0.3412	–	–	–	0.68	0.4062	–	–	–
	No arrhythmia	1497 (1501.64)	152 (147.36)	82.07	84.92										
PE	PE	28 (36.43)	12 (3.57)	1.54	6.70	22.25	** < 0.00001**	**4.609**	2.301	9.231	23.33	** < 0.00001**	**4.772**	2.377	9.579
	No PE	1796 (1787.57)	167 (175.43)	98.46	93.30										
AHF	AHF	155 (158.45)	19 (15.55)	8.50	10.61	0.92	0.3373	–	–	–	0.94	0.3330	–	–	–
	No AHF	1669 (1665.55)	160 (163.45)	91.50	89.39										
Other CVDs	Other CVDs	499 (501.76)	52 (49.24)	27.36	29.05	0.23	0.6284	–	–	–	0.32	0.5703	–	–	–
	ACS/arrhythmia/PE/AHF	1325 (1322.24)	127 (129.76)	72.64	70.95										

Subset distribution (sex: female, N = 767)															
Variable	Class	SARS-Cov-2: negative	SARS-Cov-2: positive	SARS-Cov-2: negative	SARS-Cov-2: positive	χ^2^	p	OR	OR {-95% CI}	OR {95% CI}					
		Count (n)	Count (n)	Frequency (%)	Frequency (%)										
*ACS*	ACS	263 (256.62)	15 (21.38)	37.15	25.42	3.24	0.0719	–	–	–					
	No ACS	445 (451.38)	44 (37.62)	62.85	74.58										
*Arrhythmia*	Arrhythmia	154 (154.15)	13 (12.85)	21.75	22.03	0.0026	0.9597	–	–	–					
	No arrhythmia	554 (553.85)	46 (46.15)	78.25	77.97										
*PE*	PE	14 (18.46)	6 (1.54)	1.98	10.17	14.39	**0.0002**	**5.612**	2.072	15.199					
	No PE	694 (689.54)	53 (57.46)	98.02	89.83										
*AHF*	AHF	58 (62.77)	10 (5.23)	8.19	16.95	5.17	**0.0230**	**2.287**	1.101	4.752					
	No AHF	650 (645.23)	49 (53.77)	91.81	83.05										
*Other CVDs*	Other CVDs	219 (216.0)	15 (18.0)	30.93	25.42	0.78	0.3773	–	–	–					
	ACS/arrhythmia/PE/AHF	489 (492.0)	44 (41.0)	69.07	74.58										

Subset distribution (sex: male, N = 1236)															
Variable	Class	SARS-Cov-2: negative	SARS-Cov-2: positive	SARS-Cov-2: negative	SARS-Cov-2: positive	χ^2^	*p*	OR	OR {− 95% CI}	OR {95% CI}					
		Count (n)	Count (n)	Frequency (%)	Frequency (%)										
*ACS*	ACS	552 (547.17)	54 (58.83)	49.46	45.00	0.86	0.3528	–	–	–					
	No ACS	564 (568.83)	66 (61.17)	50.54	55.00										
*Arrhythmia*	Arrhythmia	173 (168.84)	14 (18.16)	15.50	11.67	1.24	0.2653	–	–	–					
	No arrhythmia	943 (947.16)	106 (101.84)	84.50	88.33										
*PE*	PE	14 (18.06)	6 (1.94)	1.25	5.00	9.55	**0.0020**	**4.143**	1.562	10.990					
	No PE	1102 (1097.94)	114 (118.06)	98.75	95.00										
*AHF*	AHF	97 (95.71)	9 (10.29)	8.69	7.50	0.20	0.6578	–	–	–					
	No AHF	1019 (1020.29)	111 (109.71)	91.31	92.50										
*Other CVDs*	Other CVDs	280 (286.22)	37 (30.78)	25.09	30.83	1.87	0.1710	–	–	–					
	ACS/arrhythmia/PE/AHF	836 (829.78)	83 (89.22)	74.91	69.17										

Numbers in ‘()’ brackets indicate expected (estimated) counts. Significant odds ratios (ORs) are marked in bold.

Only female patients showed a significant difference in the incidence of AHF in the context of SARS-CoV2 test results. The odds for AHF in COVID-positive women were approximately 2.2871-fold higher, compared to the odds in COVID-negative women (frequencies: 16.95% vs. 8.19%). It should be emphasized that this observation could not be generalized to describe the entire population (regardless of sex), as the OR computed in the whole population sample, corrected for stratification, was insignificant (p ≈ 0.3330).

Symptoms of heart failure during ACS were significantly more frequent in COVID-positive male patients, compared to their COVID-negative counterparts (frequencies: 36.73% vs. 14.62%; OR ≈ 3.3897; Table 6). Conversely in this context, women showed insignificant differences in frequency (*p* ≈ 0.9536; Table 6); virtually, this observation could have been affected by small counts reported among COVID-positive women. The hypothesis that symptoms of heart failure are more frequent among all COVID-positive patients with ACS (regardless of sex) remained significant after adjusting for stratification. The overall odds ratio describing this incidence in the whole population sample was approximately 2.7082 (Table 6).

**Table 6:** Distribution of detailed CVD types in the context of SARS-Cov-test and sex.

Overall distribution (regardless of sex)															
Variable	Class	SARS-Cov-2: negative	SARS-Cov-2: positive	SARS-Cov-2: negative	SARS-Cov-2: positive	No stratification corrections					Generalized CMH (adjusted for stratification by sex)				
		Count (n)	Count (n)	Frequency (%)	Frequency (%)	χ^2^	*p*	OR	OR {− 95% CI}	OR {95% CI}	χ^2^	*p*	OR	OR {− 95% CI}	OR {95% CI}
ACS: type	NSTEMI	274 (280.51)	30 (23.49)	34.77	45.45	3.10	0.2120	–	–	–	3.67	0.1575	–	–	–
	STEMI	410 (404.15)	28 (33.85)	52.03	42.42										
	Unstable angina	104 (103.34)	8 (8.66)	13.20	12.12										
ACS: heart failure	Symptoms present	116 (126.2)	21 (10.8)	15.76	33.33	12.61	**0.0004**	**2.672**	1.526	4.679	12.96	**0.0003**	**2.708**	1.546	4.743
	No symptoms	620 (609.8)	42 (52.2)	84.24	66.67										
Arrhythmia: type	Atrial	145 (144.75)	13 (13.25)	49.15	48.15	0.08	0.7757	–	–	–	3.20	0.2016	–	–	–
	Ventricular	41 (43.98)	7 (4.02)	13.90	25.93										
	Heart block, bradycardia	109 (106.27)	7 (9.73)	36.95	25.93										
Arrhythmia: heart failure	Symptoms present	75 (73.98)	4 (5.02)	28.30	22.22	0.31	0.5779	–	–	–	0.28	0.5949	–	–	–
	No symptoms	190 (191.02)	14 (12.98)	71.70	77.78										
PE: heart failure	Symptoms present	9 (7.43)	2 (3.57)	36.00	16.67	0.67	0.4121	–	–	–	1.40	0.2368	–	–	–
	No symptoms	16 (17.57)	10 (8.43)	64.00	83.33										
AHF: type	CHF decompensation	95 (95.78)	10 (9.22)	70.37	76.92	0.031	0.8594	–	–	–	0.40	0.5267	–	–	–
	De novo	40 (39.22)	3 (3.78)	29.63	23.08										

Subset distribution (sex: female)															
Variable	Class	SARS-Cov-2: negative	SARS-Cov-2: positive	SARS-Cov-2: negative	SARS-Cov-2: positive	χ^2^	p	OR	OR {− 95% CI}	OR {95% CI}					
		Count (n)	Count (n)	Frequency (%)	Frequency (%)										
ACS: type	NSTEMI	106 (105.73)	6 (6.27)	41.90	40.00	0.88	0.6427	–	–	–					
	STEMI	111 (112.34)	8 (6.66)	43.87	53.33										
	Unstable angina	36 (34.93)	1 (2.07)	14.23	6.67										
ACS: heart failure	Symptoms present	42 (42.42)	3 (2.58)	18.26	21.43	0.00	0.9536	1.221	0.326	4.569					
	No symptoms	188 (187.58)	11 (11.42)	81.74	78.57										
Arrhythmia: type	Atrial	81 (80.76)	7 (7.24)	55.86	53.85	5.40	0.0672	–	–	–					
	Ventricular	15 (17.44)	4 (1.56)	10.34	30.77										
	Heart block, bradycardia	49 (46.8)	2 (4.2)	33.79	15.38										
Arrhythmia: heart failure	Symptoms present	28 (26.13)	0 (1.87)	22.22	0.00	1.35	0.2448	–	–	–					
	No symptoms	98 (99.87)	9 (7.13)	77.78	100.00										
PE: heart failure	Symptoms present	5 (4.0)	1 (2.0)	41.67	16.67	0.28	0.5959	–	–	–					
	No symptoms	7 (8.0)	5 (4.0)	58.33	83.33										
AHF: type	CHF decompensation	32 (31.5)	4 (4.5)	65.31	57.14	< 0.00001	1.0000	–	–	–					
	De novo	17 (17.5)	3 (2.5)	34.69	42.86										

Subset distribution (sex: male)															
Variable	Class	SARS-Cov-2: negative	SARS-Cov-2: positive	SARS-Cov-2: negative	SARS-Cov-2: positive	χ^2^	*p*	OR	OR {− 95% CI}	OR {95% CI}					
		Count [n]	Count [n]	Frequency [%]	Frequency [%]										
ACS: type	NSTEMI	168 (175.29)	24 (16.71)	31.40	47.06	5.90	0.0524	–	–	–					
	STEMI	299 (291.24)	20 (27.76)	55.89	39.22										
	Unstable angina	68 (68.47)	7 (6.53)	12.71	13.73										
ACS: heart failure	Symptoms present	74 (83.88)	18 (8.12)	14.62	36.73	15.79	**0.0001**	**3.390**	1.804	6.371					
	No symptoms	432 (422.12)	31 (40.88)	85.38	63.27										
Arrhythmia: type	Atrial	64 (64.02)	6 (5.98)	42.67	42.86	0.18	0.9135	–	–	–					
	Ventricular	26 (26.52)	3 (2.48)	17.33	21.43										
	Heart block, bradycardia	60 (59.45)	5 (5.55)	40.00	35.71										
Arrhythmia: heart failure	Symptoms present	47 (47.9)	4 (3.1)	33.81	44.44	0.083	0.7729	–	–	–					
	No symptoms	92 (91.1)	5 (5.9)	66.19	55.56										
PE: heart failure	Symptoms present	4 (3.42)	1 (1.58)	30.77	16.67	0.0078	0.9245	–	–	–					
	No symptoms	9 (9.58)	5 (4.42)	69.23	83.33										
AHF: type	CHF decompensation	63 (64.5)	6 (4.5)	73.26	100.00	0.95	0.3295	–	–	–					
	De novo	23 (21.5)	0 (1.5)	26.74	0.00										

Numbers in ‘()’ brackets indicate expected (estimated) counts. Significant odds ratios (ORs) are marked in bold.

## Discussion

In this multi-centre analysis across 5 European countries, we examined the impact of the COVID-19 pandemic on the distributions of selected CVDs in emergency cardiac departments. Our findings revealed significant changes in admission patterns before and during the COVID-19 pandemic. First and foremost, we observed reduced numbers of hospitalizations because of AHF, which is consistent with prior studies^8^. The relevant decline in AHF hospitalizations may be explained by fear of exposure to SARS-CoV2 in hospitals, restructured and overloaded emergency departments and unavailable ambulance services^9^. However, the obtained data indicated that de novo AHF occurred more frequently in 2020, in comparison with the deterioration of CHF. This outcome did not remain significant after adjustment associated with stratification by sex. Nevertheless, this finding is worthy of attention. Perhaps, this drop in count discloses patients’ utmost effort to avoid hospital-acquired respiratory infections, reflecting attempts to handle with exacerbation of CHF by the patients themselves. We cannot discard that a proportion of patients that did not present to the hospital due to ACS could present lately due to de novo AHF. Shaoib et al. reported an increased frequency of HF-associated deaths at home, while the numbers of HF hospitalizations dropped by 47%^10^. This observation underlines the essential role of specific self-care programs supporting CHF patients. Interestingly, dedicated artificial intelligence solutions such as a mobile app that can modify diuretic dose in response to symptoms can provide a chance to survive long enough to be admitted to the hospital^11^.

In the second part of our study, we analyzed the impact of confirmed COVID-19 infection on cardiovascular morbidity. Essentially, the distribution of all CVD differed significantly in patients with positive and negative COVID-19 tests, regardless of sex. The SARS-CoV2 virus initiates various pathophysiological pathways, including multi-organ injury, vascular wall injury and activation of inflammatory and coagulation pathways^12^, which may explain the multiple clinical manifestations^13^. The most prominent difference is seen in terms of the incidence of PE; the increase in risk of thrombotic events during COVID-19 is well described^14^. Our data shows that COVID-19 particularly increased PE risk in women, in comparison with COVID-negative counterparts. These results may suggest that COVID-19 initiates sex-specific pathways, which causes greater thrombotic activation in women than in men. Noteworthy, these findings are contradictory to prior studies which suggested higher incidence of thrombotic events in men^15^.

We have noted that ACS patients who were COVID-19 positive more frequently had symptoms of heart failure. This can stem from various factors. Firstly, COVID-19 and heart failure have partially overlapping symptomatology e.g. occurrence of dyspnea. Therefore, patients suffering from either ACS or COVID-19 are more prone to present with exacerbated respiratory symptoms. Furthermore, the aforementioned COVID-19 driven thrombotic activation may lead to more massive ACS, resulting in greater coronary artery occlusion, thus—more symptomatic manifestation of the disease. The trend toward more exacerbated myocardial infarction during COVID-19 could be seen based on our data, since there was a higher proportion of STEMI in COVID-19 patients compared to patients not infected with COVID-19 (although this finding was not statistically significant). This trend was not observed among patients suffering from NSTEMI and UA. Finally, neurohormonal activation and reduced oxygen saturations accompanying COVID-19 may deepen the cardiovascular decompensation caused by the ACS^16^.

The change in cardiovascular disease patterns and sex-specific changes in the pattern of cardiovascular events in periods with different seek for medical care, should be acknowledged and possibly managed with digital health solutions and/for further information for both medical personell and patients to better stratify diseases avoid disease severity.

## Limitations

Several limitations of our study should be mentioned. First, we did not collect any longer-term follow-up or mortality information and our data focused in specific departments of each centre. Reports of mortality rate, recurrent myocardial infarction or PE events could provide detailed insight into long-term COVID-19 complications among cardiac patients. Moreover, the studied population although large and prospective, was characterized by limited data collection, for example it did not include data regarding the disease severity or the history of other chronic diseases affecting other organ systems. As a consequence, comprehensive multi-organ disease characteristics of participants were not considered. Finally, our data were gathered in the first phase of the COVID-19 pandemic. Contemporary SARS-CoV-2 strains, the impact of vaccination and treatments such as steroids, anticoagulants and antivirals could impact of future clinical manifestations and cardiovascular morbidity.

## Conclusions

The COVID-19 pandemic had a significant influence on cardiovascular morbidity in terms of: illness distribution, severity and hospital admissions (particularly in cases of heart failure). One of the most visible trends was the higher frequency of acute thromboembolic events such as PE or STEMI and a greater tendency to develop HF during ACS. Awareness of higher cardiovascular risk in this population, especially with pre-existing cardiac comorbidities, is crucial for the early management of SARS-CoV-2 exposed patients. Simultaneously, we observed a concomitant reduction in heart failure admissions. The management of HF deterioration in times of reduced access to acute medical services poses a future challenge. Telehealth interventions via dedicated mobile apps or video consultations could be utilized to maintain patients suffering from CVDs in a stable condition.

## Data Availability

The data which support the findings of this study are available from the Institute of Heart Diseases, Wroclaw Medical University (Wroclaw, Poland) but restrictions apply to the availability of these data, which were used under license for the current study, and so are not publicly available. Data are however available from the authors upon reasonable request and with permission of the Third Parties which participated in this study (excluding for the Department of Medical Biochemistry, Wroclaw Medical University, Wroclaw, Poland). If anyone wishes to request data from this study, should contact the corresponding author at the e-mail address: dr.piotr.gajewski@gmail.com.
